# A co-rotational formulation for quasi-steady aerodynamic nonlinear analysis of frame structures

**DOI:** 10.1016/j.heliyon.2023.e19990

**Published:** 2023-09-14

**Authors:** Mauricio C. Vanzulli, Jorge M. Pérez Zerpa

**Affiliations:** a Instituto de Ingeniería Mecánica y Producción Industrial, Facultad de Ingeniería, Universidad de la República, Montevideo, Uruguay; b Instituto de Estructuras y Transporte, Facultad de Ingeniería, Universidad de la República, Montevideo, Uruguay

**Keywords:** Co-rotational formulation, Nonlinear dynamics, Quasi-steady theory, Finite element method

## Abstract

The design of structures submitted to aerodynamic loads usually requires the development of specific computational models considering fluid-structure interactions. Models using structural frame elements are developed in several relevant applications such as, the design of advanced aircraft wings, wind turbine blades or power transmission lines. In the case of flexible frame structures submitted to fluid flows, the computation of inertial and aerodynamic forces for large displacements and rotations is a challenging task. In this article, we present a novel formulation for the efficient computation of aerodynamic forces in frame structures, coupling the co-rotational framework with the quasi-steady theory. A numerical procedure is provided considering a tangent matrix for the aerodynamic forces. This formulation is implemented in the open-source library ONSAS, allowing users to reproduce the results or solve other frame nonlinear dynamic problems. The proposed formulation and its implementation are validated through the resolution of four numerical examples. The formulation and the numerical procedure proposed efficiently provide accurate solutions for these challenging problems with large displacements and rotations.

## Highlights

• An improved co-rotational formulation for computing aerodynamic forces including pitch moment in frame structures.
• The quasi-steady theory is considered for computing the aerodynamic forces.
• A consistent co-rotational formulation for nonlinear dynamics is considered.
• The proposed formulation was implemented within the open-source tool ONSAS.m.
• All the scripts used to obtain the presented results are publicly available.

## 1 Introduction

Nonlinear structural dynamic problems are formulated in a vast and diverse set of applications such as: developing new wind turbines systems [2], [39], designing suspended bridges or aircraft wings [47], [6], predicting failures in power transmission lines [41], reducing fruit production losses [8] or even studying the movement of aquatic plants [20]. In all of these applications, structures can be modeled using frame elements, and are also submitted to loads caused by the interaction with fluid flows. The development of an efficient and accurate numerical method for the resolution of this type of problems is the main motivation of this article.

Structural design standards have a limited range of application, and are not applicable to most of problems mentioned above [14], [38]. Given this limitation, alternative approaches are mainly based on experimental tests [8] or numerical simulations [42]. Experimental tests might be expensive and/or challenging to design, therefore, new numerical methods for accurate structural dynamics simulations are actively developed [17].

The Finite Element Method (FEM) [48] has become the gold-standard for computational modeling in structural analysis in numerous disciplines. For frame structures, the co-rotational approach has shown several advantages, including a more versatile and less intricate mathematical formulation [10]. This approach is based on splitting the element deformation in: one rigid movement and one local deformation [5]. Different co-rotational formulations were developed for solving static [31], stability [4] or dynamic [25] structural analysis problems. In [30] a co-rotational formulation using a projector matrix is presented and applied to nonlinear solid analysis. A consistent formulation for three-dimensional nonlinear dynamic analysis of frame structures was presented [26], allowing to accurately simulate deformations with large displacements and rotations using a reduced number of elements. In [44] it is shown that, for structures submitted to large rotations, the consistent formulation is considerably more accurate and efficient than the lumped mass approach.

In the last decades different frame analysis formulations were used for the mentioned applications of interest. In [13], a three-dimensional nonlinear three-node isoparametric element is used for modeling the movement of overhead transmission lines, considering a consistent mass matrix for linear inertial terms. With the same purpose, in [40] a three-dimensional linear frame element was used to simulate cable elements. In [28], a formulation considering nonlinear internal forces with a lumped mass matrix for linear inertial terms was used for modeling wind turbine blades. In [39] a vorticity wind turbine was modeled using the discrete element method concluding that, large displacements must be considered to emulate states of maximum output power. Regarding the nonlinear geometric analysis of wind turbine blades, the linearized equations of motion were solved in [16], obtaining a good level of agreement between the *exact beam theory* and formulations using shell elements. In [11], [29], static co-rotational formulations were used to simulate morphing or highly flexible wings, highlighting the computational efficiency to validate experimental results. However, the performance of a formulation considering consistent inertial terms and aerodynamic forces using the co-rotational framework, has not been reported.

Regarding the availability of software for the numerical resolution of these problems, three specific tools can be mentioned: RIFLEX, FAST and HAWC2. RIFLEX is a proprietary software developed for fluid-structure interaction problems. It uses a co-rotational approach for modeling frame elements, a linear consistent mass matrix, a Rayleigh damping matrix and allows to compute mass, shear and elastic centers [9], [12]. FAST is a modular open-source framework for fluid structure numerical simulations. This software uses beam elements based on the *exact beam theory* and a Timoshenko mass matrix [45], [32]. HAWC2 is a proprietary software for aeroelastic simulation of wind turbines, developed using linear anisotropic Timoshenko beam elements [24].

In [19] and [21], Euler-Bernoulli and Kirchhoff nonlinear beam formulations were used to simulate the deformation of flexible structures submitted to drag and lift forces. The deformation of thin elastic blades was studied in [27], concluding that inertial effects have a significant impact on the numerical results of the model. Furthermore, in [33] a nonlinear aeroelastic study of wings was conducted concluding that deformation is the dominant nonlinearity for long slender wings.

In [35] the *quasi-steady theory* and an equivalent beam model with a lumped mass matrix were employed to analyze galloping effects on buildings. In [18] the co-rotational framework is introduced for the computation of aerodynamic forces, neglecting the pitch torsional moment, and a linear lumped mass matrix approach is used for the inertial effects. The tangent matrices of the aerodynamic forces vector were also neglected in the iterative numerical scheme applied. Therefore, there is still a research gap in quantifying the benefits of using a consistent inertial formulation and the pitch moment.

In this work, we present a novel unified formulation for consistent co-rotational analysis of frame structures submitted to nonlinear aerodynamic forces. For the first time, the fluid interaction effect is included by considering the *quasi-steady theory*, a consistent co-rotational formulation and the Principle of Virtual Work. In particular, in contrast with [18], in the proposed formulation the aerodynamic pitch moment is not neglected. In addition, our results showed that considering the aerodynamic stiffness tangent matrix, which is not usually computed in the literature, improves the accuracy of the solution in static analysis problems.

The proposed formulation is implemented in the open-source structural analysis solver ONSAS [34], providing a new tool for the scientific community. We perform numerical analyses for different flow conditions, cross-sections and magnitudes of displacements and rotations, studying changes in mesh sizes and number of Gauss numerical integration points. The formulation and its implementation are tested through the resolution of four numerical examples. In the first example the implementation is validated using the open-source tool from [19]. All the scripts used in the numerical examples, are publicly available allowing any user to automatically reproduce the results presented.

This article is organized as follows. In Section 2 the basic concepts of the co-rotational framework are described. In Section 3, the proposed formulation is presented, with a corresponding numerical procedure for the resolution of the balance equations. In Section 4 the numerical results obtained are presented, and in Section 5, the conclusions obtained are described.

## 2 Preliminaries

In this section, the fundamental concepts of the co-rotational frame analysis approach are described. The main kinematic identities and the internal and inertial forces are briefly presented considering [4], [26].

### 2.1 Co-rotational kinematics

The main concepts behind the co-rotational approach are the use of different systems of coordinates and the application of the Principle of Virtual Work. Given a two-node frame element and two systems of coordinates (global and local), a vector of generalized nodal displacements **d** can be represented in the global system of coordinates as $d_{g}$, and in the local system as $d_{\ell }$. The virtual work of a set of nodal forces **f** is the same in the local or the global system of coordinates, as it is presented in Equation (1):

(1) $${\left(\delta d_{\ell }\right)}^{T}f_{\ell }={\left(\delta d_{g}\right)}^{T}f_{g},$$

for any vector of virtual displacements δd.

**Figure 1:**
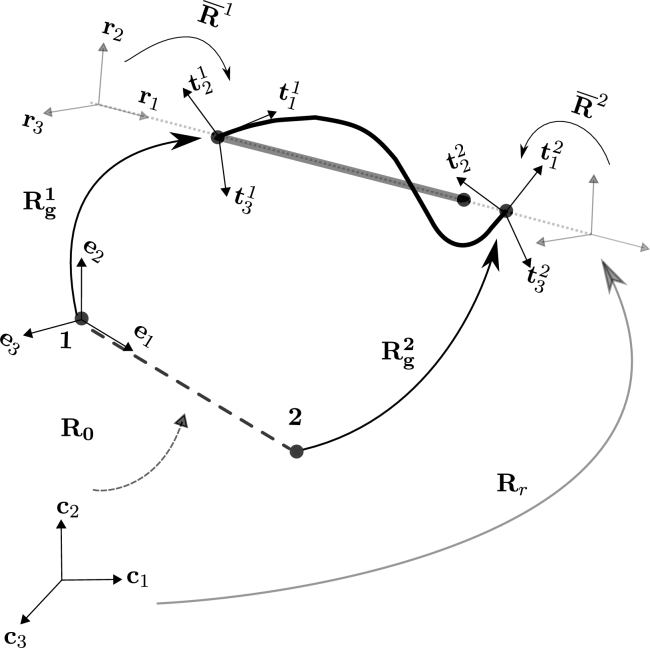
Diagram of the co-rotational framework: reference configuration (dashed line), rigid-rotation configuration (gray solid line) and total-deformed configuration (black solid curve).

In the co-rotational approach three configurations are defined as shown in Fig. 1: a reference configuration, a rigid-rotation configuration and the total-deformation configuration. As it is shown, four systems of coordinates are defined: $\left\{c_{i}\right\}$, $\left\{e_{i}\right\}$, $\left\{r_{i}\right\}$ and $\left\{t_{i}\right\}$, corresponding to the canonical, reference, rigid-rotation and total-deformed configurations, respectively. Orthogonal matrices $R_{0}$, $R_{g}$, $R_{r}$ and $\bar{R}$ can also be defined as shown in Fig. 1, to rotate the base vectors of these systems of coordinates.

**Figure 2:**
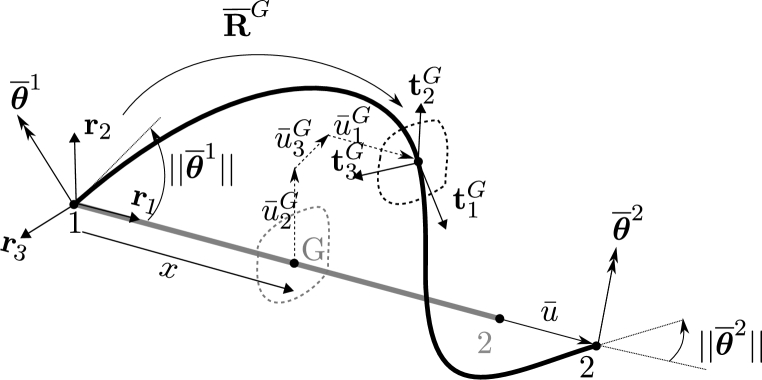
Local displacements from rigid-rotation to total-deformed configuration.

The column vector of nodal displacements written in the canonical system $\left\{c_{i}\right\}$ is denoted as $d_{g}={\left[{\left(u^{1}\right)}^{T},{\left(w^{1}\right)}^{T},{\left(u^{2}\right)}^{T},{\left(w^{2}\right)}^{T}\right]}^{T}$, where $u^{i}$ and $w^{i}$ are the column vectors of linear displacements and rotations, respectively, of node *i*. In the co-rotational approach, the displacements of the element are also written considering the system of coordinates $\left\{r_{i}\right\}$, where the local extension and the nodal rotations are grouped as $d_{\ell }={\left[\bar{u},{\left({\bar{\theta }}^{1}\right)}^{T},{\left({\bar{\theta }}^{2}\right)}^{T}\right]}^{T}$. The extension is given by $\bar{u}=l_{n}-l_{0}$ where $l_{n}$ and $l_{0}$ are the deformed and reference lengths of the element, and the local rotations are given by the vectors ${\bar{\theta }}^{i}$ as it is shown in Fig. 2.

In order to apply the Principle of Virtual Work, the vectors of variations of the generalized displacements δd need to be written in the same system of coordinates. The variation of the local extension verifies Equation (2):

(2) $$\delta \bar{u}=r\;\delta d_{g},\;r=[-r_{1}^{T}\;0_{1\times 3}\;r_{1}^{T}\;0_{1\times 3}],$$

and for the vectors of rotations Equation (3):

(3) $$\left[\begin{array}{c} \delta {\bar{\theta }}^{1}\\ \delta {\bar{\theta }}^{2} \end{array}\right]=PE^{T}\delta d_{g},\;P=\left[\begin{array}{cccc} 0_{3\times 3} & I & 0_{3\times 3} & 0_{3\times 3}\\ 0_{3\times 3} & 0_{3\times 3} & 0_{3\times 3} & I \end{array}\right]-\left[\begin{array}{c} G\\ G \end{array}\right],$$

where $0_{i\times j}$ represents a matrix of zeros with *i* rows and *j* columns (the sub-indexes are omitted for the 3×3 case), **E** is a matrix given by Equation (4):

(4) $$E=\left[\begin{array}{cccc} R_{r} & 0 & 0 & 0\\ 0 & R_{r} & 0 & 0\\ 0 & 0 & R_{r} & 0\\ 0 & 0 & 0 & R_{r} \end{array}\right],$$

and **G** is a matrix given by Equation (5):

(5) $$G=\left[\begin{array}{cccccccccccc} 0\; & 0\; & \frac{p_{1}}{p_{2}l_{n}}\; & \frac{p_{12}}{2p_{2}}\; & -\frac{p_{11}}{2p_{2}}\; & 0\; & 0\; & 0\; & -1/l_{n}\; & 0\; & 0\; & 0\\ 0\; & 0\; & 1/l_{n}\; & 0\; & 0\; & 0\; & 0\; & 0\; & -\frac{p_{1}}{p_{2}l_{n}}\; & \frac{p_{2}}{2p_{2}}\; & -\frac{p_{21}}{2p_{2}}\; & 0\\ 0\; & -1/l_{n}\; & 0\; & 0\; & 0\; & 0\; & 0\; & 1/l_{n}\; & 0\; & 0\; & 0\; & 0 \end{array}\right],$$

with $p_{ij}$ being the *j*-th entry of the vector $p_{i}$ is defined in Equation (6):

(6) $$p_{i}=R_{g}^{i}R_{0}{\left[0,1,0\right]}^{T}\;i=1,2,$$

and $p_{j}$ being the *j*-th entry of the vector **p** defined by $p=\frac{1}{2}(p_{1}+p_{2})$.

For a cross-section located at the position *x*, as shown in Fig. 2, with centroid *G* and deformed base $\left\{t_{i}^{G}\right\}$, the variations of the displacements and rotations can also be written in local and global systems, using Equations (7) a) and b):

(7) $$\text{a) }\left[\begin{array}{c} 0\\ {\overset{¯}{u}}_{2}^{G}\\ {\overset{¯}{u}}_{3}^{G} \end{array}\right]=P_{1}\left[\begin{array}{c} {\bar{\theta }}^{1}\\ {\bar{\theta }}^{2} \end{array}\right],\;\text{b)}\;P_{1}=\left[\begin{array}{cccccc} 0 & 0 & 0 & 0 & 0 & 0\\ 0 & 0 & N_{3} & 0 & 0 & N_{4}\\ 0 & -N_{3} & 0 & 0 & -N_{4} & 0 \end{array}\right],$$

and Equations (8) a) and b):

(8) $$\text{a) }\left[\begin{array}{c} {\overset{¯}{\theta }}_{1}^{G}\\ {\overset{¯}{\theta }}_{2}^{G}\\ {\overset{¯}{\theta }}_{3}^{G} \end{array}\right]=P_{2}\left[\begin{array}{c} {\bar{\theta }}^{1}\\ {\bar{\theta }}^{2} \end{array}\right],\;\text{b)}\;P_{2}=\left[\begin{array}{cccccc} N_{1} & 0 & 0 & N_{2} & 0 & 0\\ 0 & N_{5} & 0 & 0 & N_{6} & 0\\ 0 & 0 & N_{5} & 0 & 0 & N_{6} \end{array}\right],$$

where $N_{1}$ and $N_{2}$ are the linear interpolation functions (for axial displacement) and $N_{3}$, $N_{4}$, $N_{5}$ and $N_{6}$ are Hermite interpolation functions (for bending).

The position of *G* in canonical coordinates can be written as:

(9) $$OG=N_{1}(x^{1}+u^{1})+N_{2}(x^{2}+u^{2})+R_{r}u_{\ell },$$

where $u_{\ell }$ are the local transverse displacements. Considering Equation (9), the variations of the displacement and rotation of the point *G* can be written as:

(10) $$\text{a) }\delta u=R_{r}H_{1}E^{T}\delta d_{g},\;\text{and}\;\text{b) }\delta w=R_{r}H_{2}E^{T}\delta d_{g},$$

respectively, where $H_{2}=P_{2}P+G^{T}$ and $H_{1}=N+P_{1}P-\overset{˜}{u_{\ell }}G^{T}$, with $\overset{˜}{u_{\ell }}$ being the skew operator associated with the vector $u_{\ell }$.

Finally, velocities and accelerations can be obtained using Equation (11):

(11) $$\overset{˙}{u}=R_{r}H_{1}E^{T}\overset{˙}{d_{g}},\;\overset{¨}{u}=R_{r}H_{1}E^{T}\overset{¨}{d_{g}}+R_{r}C_{1}E^{T}\overset{˙}{d_{g}},\;\overset{˙}{w}=R_{r}H_{2}E^{T}\overset{˙}{d_{g}},\;\overset{¨}{w}=R_{r}H_{2}E^{T}\overset{¨}{d_{g}}+R_{r}C_{2}E^{T}\overset{˙}{d_{g}},$$

where $C_{i}=\overset{˜}{w_{r}^{e}}H_{i}+\overset{˙}{H_{i}}-H_{i}E_{t}$ and $w_{r}^{e}=GE^{T}\overset{˙}{d_{g}}$.

### 2.2 Internal and inertial forces

The expressions of the elemental internal and inertial forces in global coordinates can be obtained using the Principle of Virtual Work. Considering Equation (1) for the internal forces, and substituting the relations presented in Equations (2) and (3), we obtain Equation (12):

(12) $$\delta d_{g}^{T}f_{g}^{int}=\delta d_{g}^{T}\left[\begin{array}{cc} r^{T} & EP^{T} \end{array}\right]f_{\ell }^{int}.$$

This identity is valid for any virtual displacement $\delta d_{g}$, thus we obtain the Equation (13):

(13) $$f_{g}^{int}=\left[\begin{array}{cc} r^{T} & EP^{T} \end{array}\right]f_{\ell }^{int},$$

where $f_{\ell }^{int}$ is the known vector of internal forces $f_{\ell }^{int}=\left[f_{a\ell }\;\;{\left(m_{\ell }^{1}\right)}^{T}\;{\left(m_{\ell }^{2}\right)}^{T}\right]$, with normal force and bending moments, given by a linear constitutive behavior.

For the inertial term, the kinematic energy *K* of the element is written as:

(14) $$\text{K}=\frac{1}{2}\int_{l_{0}}\rho {\overset{˙}{u}}^{T}A\overset{˙}{u}+\rho {\overset{˙}{w}}^{T}I\overset{˙}{w}\;dl_{0},$$

where *A* is the area of the cross-section, *ρ* is the density of the material and **I** is the geometric inertia tensor. Considering the variation in both members of Equation (14) it can be obtained Equation (15):

(15) $$\delta \text{K}=-\int_{l_{0}}\delta u^{T}\rho A\overset{¨}{u}+\delta w^{T}[\rho I\overset{¨}{w}+\overset{˜}{\overset{˙}{w}}\rho I\overset{¨}{w}]dl_{0}.$$

The inertial force vector of the element in global coordinates $f_{g}^{ine}$ is then defined consistently by Equation (16):

(16) $$\delta K=-{\left(f_{g}^{ine}\right)}^{T}\delta d_{g},\;\text{with}\;f_{g}^{ine}=\int_{l_{0}}\left\{H_{1}^{T}R_{r}^{T}\rho A\overset{¨}{u}+H_{2}^{T}R_{r}^{T}[\rho I\overset{¨}{w}+\overset{˜}{\overset{˙}{w}}\rho I\overset{˙}{w}]\right\}dl_{0}.$$

## 3 Methodology

In this section we present the proposed formulation for the computation of the aerodynamic forces, and describe a numerical procedure for the resolution of the governing equations.

### 3.1 Co-rotational quasi-steady aerodynamic forces

**Figure 3:**
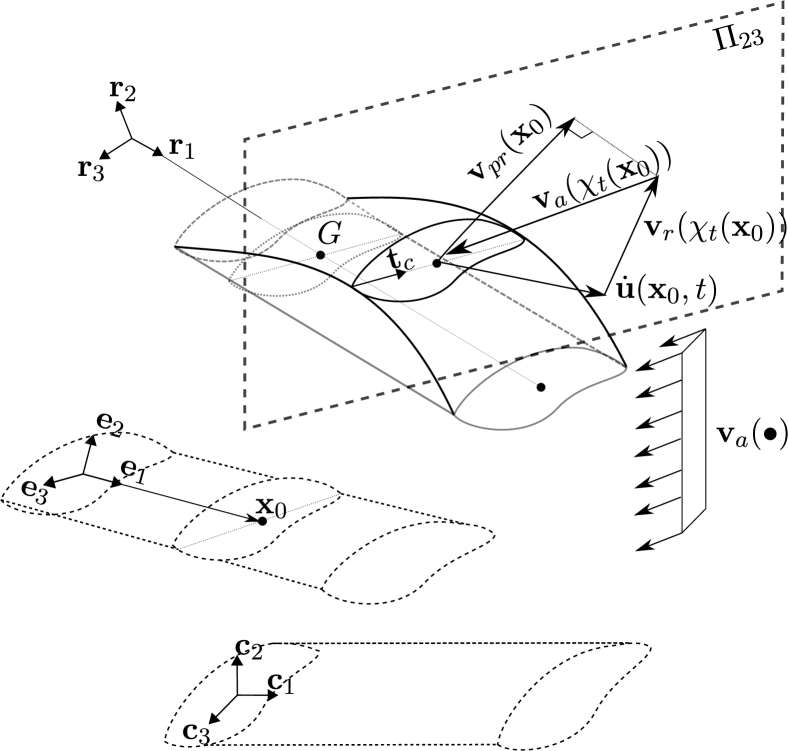
Co-rotational framework on fluid loads. Reference and initial configurations (dashed line), rigid-rotation configuration (gray solid line) and total-deformed configuration (black solid curve).

Let us consider a frame element, with uniform cross-section, submitted to a fluid flow as shown in Fig. 3. For a section located at $x_{0}$ with centroid *G*, the deformed position at time *t* is given by $x=\chi_{t}(x_{0})$. The element is submitted to forces induced by a fluid with absolute velocities given by the field $v_{a}(x,t):R^{3}\times R\to R^{3}$. The velocity of the centroid is $\overset{˙}{u}(x_{0},t)$ and the relative velocity in the deformed position is defined by:

(17) $$v_{r}(\chi_{t}(x_{0}),t)=v_{a}(\chi_{t}(x_{0}),t)-\overset{˙}{u}(x_{0},t).$$

In this definition a fundamental assumption was considered: the movement of the structure does not affect the absolute velocities of the fluid [7].

The interaction between the fluid flow and the frame element produces normal and shear stresses, that are represented by moments and forces (generalized forces) applied at the deformed position of the centroid. These forces are assumed to be uniquely defined in terms of the instantaneous position and velocity of the deformed section [18]. In particular, in this formulation, the forces are assumed to depend only on $v_{pr}$ (the projection of the relative velocity onto the plane $\Pi_{23}$ defined by $t_{2}$ and $t_{3}$) as it is shown in Fig. 3. It is assumed that the density of the fluid is considerably lower than the density of the structure, therefore the added-mass effect is neglected.

**Figure 4:**
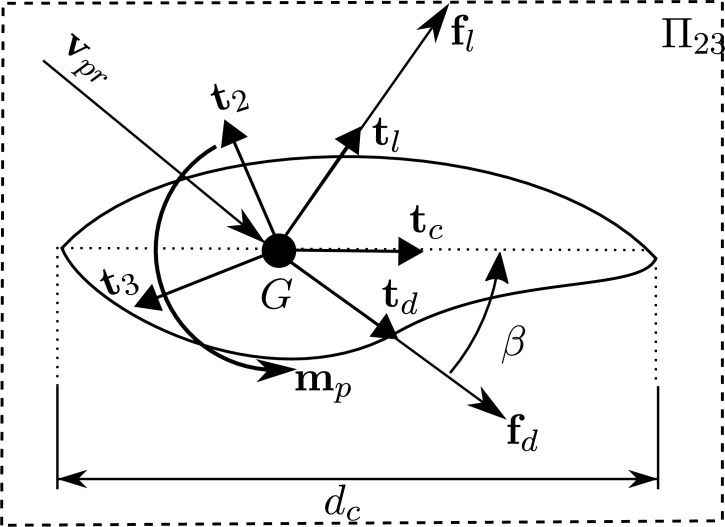
Fluid loads on a generic deformed cross-section.

The quasi-steady aerodynamic distributed forces for drag, lift and torsional moment are given by the expressions:

(18) $$\left\{ \begin{array}{cc} f_{d}\; & =\frac{1}{2}\rho_{f}d_{c}c_{d}(Re,\beta )\;{\left\|v_{pr}\right\|}^{2}t_{d},\\ f_{l}\; & =\frac{1}{2}\rho_{f}d_{c}c_{l}(Re,\beta )\;{\left\|v_{pr}\right\|}^{2}t_{l},\\ m_{p}\; & =\frac{1}{2}\rho_{f}d_{c}c_{m}(Re,\beta )\;{\left\|v_{pr}\right\|}^{2}t_{m}, \end{array}\right.$$

respectively, where $\rho_{f}$ is the density of the fluid, $d_{c}$ is the given characteristic dimension of the cross-section and $c_{d}$, $c_{l}$ and $c_{m}$ are the drag, lift and moment coefficients, determined by wind tunnel tests for different Reynolds numbers *Re* and angles of incidence *β*. The angle *β* is defined by $v_{pr}$ and the unitary chord vector of the section $t_{c}$, as shown in Fig. 4. It is remarked that drag and lift force vectors are included in the plane $\Pi_{23}$.

The vector $v_{pr}$ written in the total-deformed system of coordinates is denoted as ${\left(v_{pr}\right)}_{t}$. In the same manner, the notation (•)_**t**_ is used for any vector in this system and this sub-index is omitted for vectors in the canonical system. The expression of ${\left(v_{pr}\right)}_{t}$ is given by Equation (19):

(19) $${\left(v_{pr}\right)}_{t}={\left(v_{a}-\overset{˙}{u}\right)}_{t}-\left({\left(v_{a}-\overset{˙}{u}\right)}_{t}\cdot {\left(t_{1}\right)}_{t}\right){\left(t_{1}\right)}_{t}$$

where ${\left(t_{1}\right)}_{t}={\left[1,0,0\right]}^{T}$. Using the rotation matrices of the co-rotational framework as change of basis operators: $R_{r}{=}_{c}{\left(I\right)}_{r}$, $\bar{R}{=}_{r}{\left(I\right)}_{t}$, we can write:

(20) $${\left(v_{a}-\overset{˙}{u}\right)}_{t}={\left(R_{r}\bar{R}\right)}^{T}(v_{a}-\overset{˙}{u}).$$

Substituting Equation (20) in (19) and defining a projection operator $L_{2}$ we obtain Equation (21):

(21) $${\left(v_{pr}\right)}_{t}=L_{2}{\left(R_{r}\bar{R}\right)}^{T}(v_{a}-\overset{˙}{u}).$$

Using this we can define the unitary vectors ${\left(t_{d}\right)}_{t}$, ${\left(t_{l}\right)}_{t}$ and ${\left(t_{m}\right)}_{t}$ in Equations (22), (23) and (24):

(22) $${\left(t_{d}\right)}_{t}=\frac{{\left(v_{pr}\right)}_{t}}{\left||{\left(v_{pr}\right)}_{t}|\right|},$$

(23) $${\left(t_{l}\right)}_{t}=L_{3}{\left(t_{d}\right)}_{t},$$

(24) $${\left(t_{m}\right)}_{t}={\left(t_{1}\right)}_{t},$$

with $L_{3}=\text{exp}({\left[\pi /2,0,0\right]}^{T})$. The angle of incidence *β* verifies Equation (25):

(25) $${\left(t_{d}\right)}_{t}\cdot {\left(t_{c}\right)}_{t}=\|{\left(t_{d}\right)}_{t}\|\|{\left(t_{c}\right)}_{t}\|\cos(\beta ),$$

and considering that $t_{d}$ and $t_{c}$ are unitary we obtain the expression:

(26) $$\beta =\text{sign}\left[\left({\left(t_{d}\right)}_{t}\wedge {\left(t_{c}\right)}_{t}\right)\cdot {\left(t_{1}\right)}_{t}\right].\arccos({\left(t_{d}\right)}_{t}.{\left(t_{c}\right)}_{t}),$$

where a convention was considered as shown in Fig. 4. Using Equation (26) the angle of incidence *β* can be determined for any deformed configuration.

Substituting the identities obtained above in Equation (18) we obtain:

(27) $$\left\{ \begin{array}{ccc} {\left(f_{d}\right)}_{t}\; & =\; & \frac{1}{2}\rho_{f}d_{c}c_{d}||L_{2}{\left(R_{r}\bar{R}\right)}^{T}(v_{a}-\overset{˙}{u})||L_{2}{\left(R_{r}\bar{R}\right)}^{T}(v_{a}-\overset{˙}{u}),\\ {\left(f_{l}\right)}_{t}\; & =\; & \frac{1}{2}\rho_{f}d_{c}c_{l}||L_{2}{\left(R_{r}\bar{R}\right)}^{T}(v_{a}-\overset{˙}{u})||L_{3}L_{2}{\left(R_{r}\bar{R}\right)}^{T}(v_{a}-\overset{˙}{u}),\\ {\left(m_{p}\right)}_{t}\; & =\; & \frac{1}{2}\rho_{f}d_{c}c_{m}||L_{2}{\left(R_{r}\bar{R}\right)}^{T}(v_{a}-\overset{˙}{u})|{|}^{2}.{\left(t_{1}\right)}_{t}. \end{array}\right.$$

The virtual work corresponding to the aerodynamic forces of the element is given by Equation (28):

(28) $$\delta W_{f}=\int_{l_{0}}\left\{\delta u^{T}R_{r}\bar{R}{\left(f_{d+l}\right)}_{t}+\delta w^{T}R_{r}\bar{R}{\left(m_{p}\right)}_{t}\right\}dl_{0},$$

where $f_{d+l}$ is the sum of $f_{d}$ and $f_{l}$. Considering the vector of nodal aerodynamic generalized forces in global coordinates $f_{g}^{flu}$, the virtual work can also be written as:

(29) $$\delta W_{f}={\left(\delta d_{g}\right)}^{T}\;.\;f_{g}^{flu},$$

and substituting Equations (10) a) and b) in (28) and using Equation (29) we obtain Equation (30):

(30) $${\left(\delta d_{g}\right)}^{T}f_{g}^{flu}=\int_{l_{0}}\left\{\delta d_{g}^{T}EH_{1}^{T}R_{r}^{T}R_{r}\bar{R}{\left(f_{d+l}\right)}_{t}+\delta d_{g}^{T}EH_{2}^{T}R_{r}^{T}R_{r}\bar{R}{\left(m_{p}\right)}_{t}\right\}dl_{0}.$$

Operating we obtain Equation (31):

(31) $$f_{g}^{flu}=E\left[\int_{l_{0}}\left\{H_{1}^{T}\bar{R}{\left(f_{l+d}\right)}_{t}+H_{2}^{T}\bar{R}{\left(m_{p}\right)}_{t}\right\}dl_{0}\right],$$

and substituting Equation (27) the complete expression of the aerodynamic forces vector is obtained in Equation (32):

(32) $$f_{g}^{flu}=\frac{1}{2}\rho_{f}d_{c}E(\int_{l_{0}}\left\{H_{1}^{T}\bar{R}||L_{2}{\left(R_{r}\bar{R}\right)}^{T}(v_{a}-\overset{˙}{u})||\left[\begin{array}{c} \left(c_{d}I+c_{l}L_{3}\right) \end{array}\right]L_{2}{\left(R_{r}\bar{R}\right)}^{T}(v_{a}-\overset{˙}{u})\right\}dl_{0}\ldots \ldots +\int_{l_{0}}\left\{H_{2}^{T}\bar{R}c_{m}||L_{2}{\left(R_{r}\bar{R}\right)}^{T}(v_{a}-\overset{˙}{u})|{|}^{2}{\left(t_{1}\right)}_{t}\right\}dl_{0})$$

It is important to highlight that, in contrast with [18], in the proposed formulation the pitch moment is not neglected.

### 3.2 Balance equations and numerical resolution procedure

The governing equations are obtained by considering the virtual work for all the elements of the structure for the forces in Equations (13), (16) and (32). Additionally a vector with external forces not induced by the fluid interaction $f_{g}^{ext}$ can be added. The nonlinear system of governing equations is written in Equation (33):

(33) $$f_{g}^{ext}(t)+f_{g}^{flu}(d_{g},{\overset{˙}{d}}_{g},v_{a})-f_{g}^{int}(d_{g})-f_{g}^{ine}(d_{g},{\overset{˙}{d}}_{g},{\overset{¨}{d}}_{g})=0$$

where the arguments of the residual forces $f^{res}$ were omitted.

The numerical resolution procedure proposed consists in solving the system of nonlinear governing equations using iterative methods. For the static analysis cases the Newton-Raphson method is used, while for dynamic analysis cases the Newmark method with $\alpha_{N}=1/4$ and $\delta_{N}=1/2$ [3], and the *α*-HHT method with $\alpha_{H}=-0.05$ [26] are used. The computation of the aerodynamic and inertial force vectors is done using numerical integration.

Three different formulations are considered and used to generate the numerical results:

**F1**: In this formulation a lumped mass approach is considered for the inertial terms and the aerodynamic forces are computed using the equations presented in Section 3.1, with $c_{m}=0$ (neglecting torsional moment). Also, the tangent matrices of the aerodynamic force vector are neglected. This formulation is considered to be equivalent to the one presented in [18].
**F2**: In this formulation the consistent approach [26] is considered for the inertial terms, and the aerodynamic force vector is computed using the equations presented in Section 3.1 (without neglecting the torsional moment). The tangent matrices of the aerodynamic force vector are neglected.
**F3**: In this formulation the forces are computed as in **F2**, however for the tangent matrix the contribution of the aerodynamic forces is added. The aerodynamic stiffness matrix is computed for each frame element using a finite difference approach given by Equation (34):
  (34) $$K_{T,i}^{flu}=\frac{f_{g}^{flu}(d_{g}+he_{i},{\overset{˙}{d}}_{g},v_{a})-f_{g}^{flu}(d_{g},{\overset{˙}{d}}_{g},v_{a})}{h}\;i=1...12$$
  where $K_{T,i}^{flu}$ is the *i*-th column of the aerodynamic stiffness matrix, $h=1\times 10^{-10}$ m and $e_{i}$ is a canonical vector.

## 4 Numerical results

In this section the numerical results obtained for four problems are presented. Unless it is specified the fluid considered is air with density $\rho_{f}=1.225$ kg/m^3^, kinematic viscosity $\nu_{f}=1.5\times 10^{-5}$ m^2^/s, at 20 ^∘^C and atmospheric pressure. Regarding the elastic properties, Poisson's ratio ν=0.3 is considered for the first four examples. For all the problems, homogeneous initial conditions are considered.

All the numerical results presented can be reproduced by running scripts publicly available.^1^

The results shown were produced using a computer with a Linux OS, a 64-bit architecture, an Intel i7-11370H CPU and 16 Gb of RAM, running the implementation of the formulations in ONSAS on GNU-Octave [15]. The visualization is done using Paraview [1] and GNU-Octave.

The stopping criteria considered in all the examples are given by Equation (35):

(35) $$\frac{\left\|\Delta d_{g,s}^{k}\right\|}{\left\|d_{g,s}^{k}\right\|}⩽tol_{u}\;\text{and}\;\|\Delta f^{res,k}\|⩽tol_{r},$$

where *k* is the number of iteration and $tol_{u}$, $tol_{r}$ are scalars to be defined.

### 4.1 Example 1: drag reconfiguration of a cylindrical cantilever beam

In this example a cantilever beam submitted to a flow producing drag forces is considered. The main goal is to obtain results about the performance of the three formulations described in Section 3.2, for a problem with large displacements. The example is based on one of the problems considered in [19], where a reference solution is presented and validated with experimental data. Finally, a brief numerical study on the variation of the results for different numbers of Gauss integration points is presented.

#### 4.1.1 Problem definition

**Figure 5:**
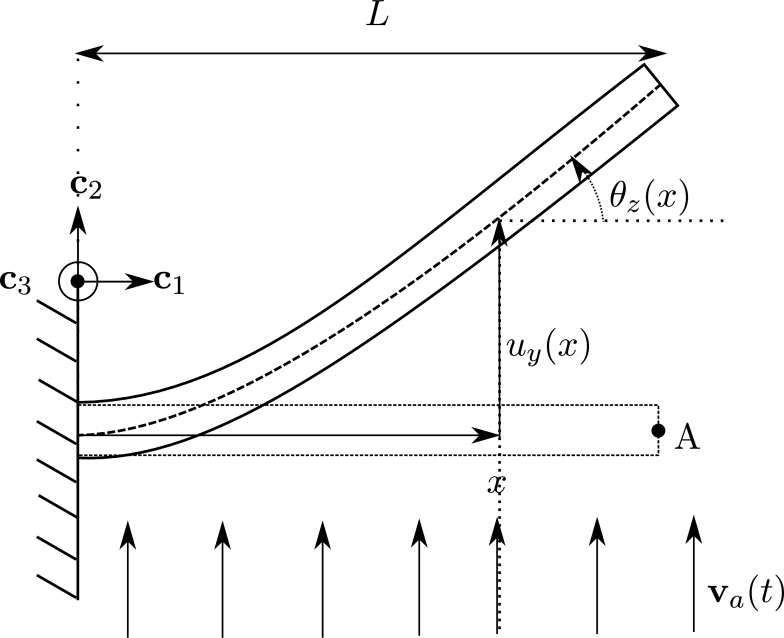
Example 1: Diagram of cantilever beam with boundary conditions and fluid flow.

The problem consists in a cantilever beam submitted to a fluid flow with uniform velocity $v_{a}(x,t)=v_{a}(t)c_{2}$, as shown in Fig. 5. The beam is clamped on the boundary at x=0 m, and the span length is L=1 m. The cross-section of the beam is circular with diameter d=1 cm, and the chord length used to compute the aerodynamic forces is $d_{c}=d$. For the material of the beam a linear elastic isotropic model is considered, with Young modulus E=30 MPa and density ρ=7000 kg/m^3^.

The fluid considered is water with density $\rho_{f}=1020$ kg/m^3^ and kinematic viscosity $\nu_{f}=10^{-6}$ m^2^/s. The values of the aerodynamic coefficients are considered from [37] as: $c_{d}=1.2$ and $c_{l}=c_{m}=0$.

#### 4.1.2 Numerical results: static case

The goal of this analysis case is to study the results obtained with formulations F1, F2 and F3 for fluid velocities $v_{a}\in \{0.005,0.012,0.029,0.072,0.176,0.432,1.057,2.588,6.336,15.512\}$ all in m/s.

**Figure 6:**
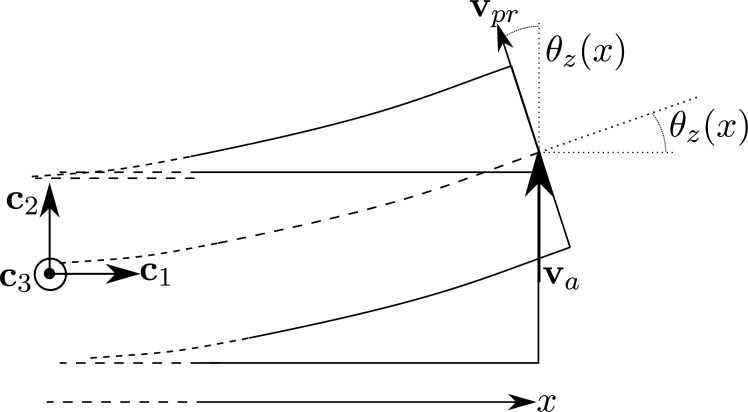
Example 1: Absolute and projected transversal velocities for the static case.

This is a static analysis case, thus the velocity of any point of the beam is $\overset{˙}{u}=0$. Substituting this in Equation (17) we obtain $v_{r}(x,t)=v_{a}(x,t)$. In Fig. 6 the absolute velocity of the fluid and its relative projected transversal component are shown, and for this analysis case, we obtain the identity:

(36) $$\left\|v_{pr}(x)\right\|=\left\|v_{a}(x)\right\|.\left|\cos\left(\theta_{z}(x)\right)\right|.$$

Equation (36) indicates that once the beam is deformed, the norm of the projected velocity decreases, and therefore the drag force decreases. This geometric nonlinearity effect is called reconfiguration. Due to this reconfiguration mechanism the drag load does not increase with the square of the fluid velocity [19]. The problem can be studied through the dimensionless Equation (37):

(37) $$c_{y}=\frac{\rho_{f}L^{3}v_{a}^{2}}{16EI_{zz}},\;R=\frac{F}{\frac{1}{2}\rho_{f}Ldc_{d}v_{a}^{2}}$$

where *F* is the global drag force towards $c_{2}$, $c_{y}$ is the Cauchy number that describes the ratio between the stiffness of the beam and the flow load and the reconfiguration number R reflects the geometric nonlinear effect by dividing the drag of the flexible beam to that of a rigid one of the same geometry.

**Figure 7:**
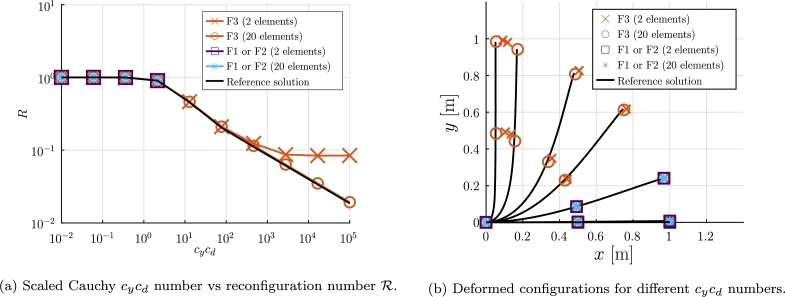
Example 1: Drag reconfiguration validation against [19].

For each velocity $v_{a}$, the numerical formulations are used to obtain the solution and the drag force *F* is computed. The N-R method is used with only force stopping criteria considering $tol_{r}=10^{-8}$. The results obtained for R (using 2 and 20 elements) are shown in Fig. 7a, and a reference solution is also obtained using the source code^2^ developed for [19].

It is observed that the three formulations match the reference solution for $v_{a}⩽0.072$ m/s. Moreover, when the aerodynamic load exhibits high geometrical nonlinearities ($v_{a}>0.072$ m/s) F3 converges while F2 and F1 do not. This demonstrates that in this case, the stiffness aerodynamic matrix is necessary to accurately reproduce the reference results. Furthermore, a larger number of elements is required to verify the reference solution accurately in cases with $v_{a}⩾1.057$ m/s.

It is reported that, for $v_{a}=0.072$ m/s and using 20 elements, the formulation F3 requires 6 times the execution time required by F1 or F2 formulations.

#### 4.1.3 Numerical results: dynamic case

In this case a nonlinear dynamic analysis is performed and the flow velocity $v_{a}$ is given by Equation (38):

(38) $$v_{a}(x,t)=\left\{ \begin{array}{cc} v_{a}\frac{t}{t_{c}}\;\; & t\in [0,t_{c}],\\ v_{a}\; & t\in (t_{c},+\infty ), \end{array}\right.$$

where $t_{c}=7$ s and two values are considered for $v_{a}$: 6.3355 m/s (Case 1), and 1.0568 m/s (Case 2). The trapezoidal Newmark numerical method is used, with time step Δt=0.05 s and the stopping criteria are $tol_{u}=1\times 10^{-10}$ and $tol_{r}=1\times 10^{-4}$.

**Figure 8:**
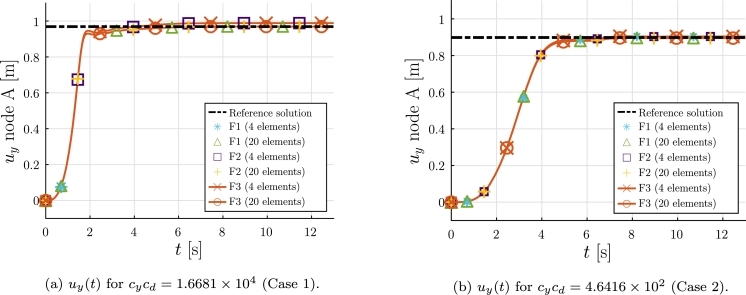
Example 1: Evolution of *u*_*y*_ displacement of node A.

The solutions obtained for the displacement $u_{y}$ of point A are shown in Fig. 8. The solutions provided by the three formulations F1, F2, and F3, using 20 elements for both cases, converge to the reference solution. Moreover the formulation F1, using 4 elements in Case 1, does not converge after t≈1.5 s.

**Figure 9:**
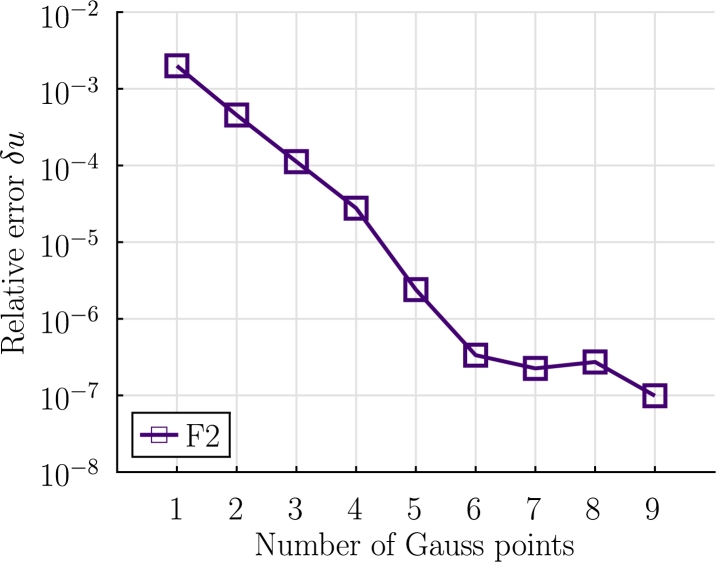
Example 1: Relative error *δ*_*u*_ for different numbers of Gauss integration points at *t* = 7 s.

In order to analyze the numerical behavior of the formulations described, a study considering different number of integration Gauss points for the formulation F2 is presented. The displacement functions obtained at time at t=7 s for different number of integration points are compared. The difference between the functions is computed considering Equation (39):

(39) $$\delta_{u}=\frac{\int_{\ell_{0}}\left|u(x_{0},t)-u_{ref}(x_{0},t)\right|dx_{0}}{\int_{\ell_{0}}\left|u_{ref}(x_{0})\right|dx_{0}},$$

where the reference solution is obtained using 20 elements and 10 Gauss integration points. The results obtained are shown in Fig. 9.

It is noted that at t=7 s a considerable curvature is present in the displacement field ($u_{y}$ of node A is ≈1 m), thus, a high nonlinearity is also present in the aerodynamic forces term. Given the results shown in Fig. 9, 4 Gauss points are considered for integrating the aerodynamic forces.

The results obtained let us conclude that the formulations considered can be used to solve the problem. For static cases, including the aerodynamic stiffness matrix is necessary to accurately solve problems with large bending deformations. For dynamic cases, the three formulations considered are able to provide accurate results when an appropriate discretization is used. Formulation F3 requires more computational time than F1 and F2, therefore it is recommended only for static analyses of structures, submitted to large deformations.

### 4.2 Example 2: simple propeller model

In this example a simple propeller submitted to lift forces is considered. This problem is used to validate the results provided by the proposed formulation in a dynamic case with large displacements and rotations.

#### 4.2.1 Problem definition

**Figure 10:**
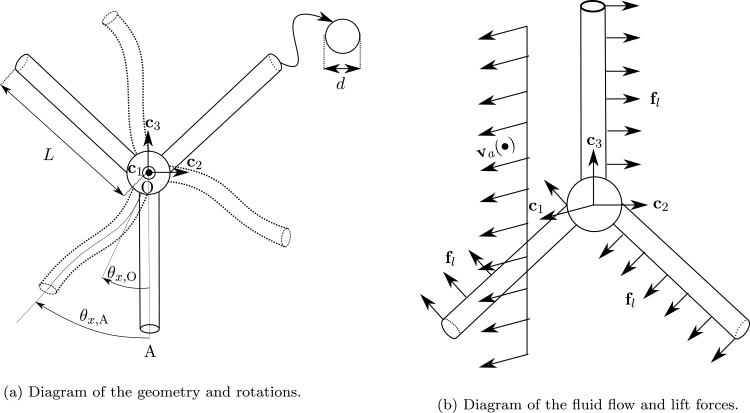
Example 2: Diagram of a simple propeller.

The problem consists in a three-blade propeller submitted to a flow with uniform velocity. Each blade has a length L=3 m and a circular cross-section with diameter d=0.1 m, as shown in Fig. 10a. Regarding the stiffness of the blades, two cases are considered: a rigid case (with analytic solution) and a flexible case, providing considerably large rotations and bending of the blades. The density ρ=6000 kg/m^3^ is considered. Regarding boundary conditions, the node O has five degrees of freedom fixed: the three displacements and rotations $\theta_{y}$ and $\theta_{z}$. The rotation $\theta_{x,\text{O}}$ is free.

A uniform flow $v_{a}=1\;c_{1}$ m/s is applied with synthetic aerodynamic coefficients $c_{l}=0.2$ and $c_{d}=c_{m}=0$. Given this, a uniform lift distributed force $f_{l}$ contained in the plane $c_{2}$-$c_{3}$ is induced, as shown in Fig. 10b. These specific settings allow us to obtain an analytic solution for the rigid case.

The *α*-HHT numerical integration method is used, with a time step increment set to Δt=1 s.

#### 4.2.2 Numerical results: rigid case

For this case, and using the value selected for $v_{a}$, it can be assumed that $\overset{˙}{u}\ll v_{a}$, thus $v_{pr}\approx v_{a}$. For the considered properties and boundary conditions, and for a Young modulus E=210 GPa, the bending deformation of the blades can be neglected, allowing to obtain an analytic solution.

Considering $\theta_{x,\text{O}}\approx \theta_{x,\text{A}}=\theta_{x}$, the angular momentum balance equation is written in Equation (40):

(40) $$\frac{1}{2}\rho_{f}c_{l}d||v_{a}|{|}_{2}^{2}\frac{L^{2}}{2}=\frac{1}{3}\rho L\pi \frac{d^{2}}{4}L^{2}\overset{¨}{\theta_{x}},$$

and using the homogeneous initial conditions, Equation (41) is obtained:

(41) $$\theta_{x}(t)=\frac{3\rho_{f}c_{l}||v_{a}|{|}_{2}^{2}}{2\rho Ld\pi }t^{2}.$$

For the numerical resolution, the tolerances $tol_{r}=10^{-6}$ and $tol_{u}=10^{-12}$ are set, and the final simulation time is $t_{f}=450$ s.

**Figure 11:**
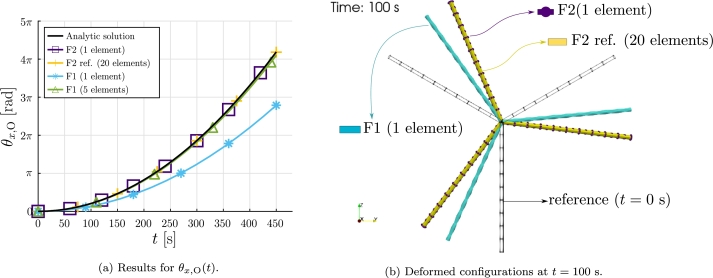
Example 2: Rigid case results.

The results obtained for $\theta_{x,O}$ are shown in Fig. 11a, where it can be observed that the analytic solution is verified with the results provided by the proposed F2 formulation, even using one element per blade. In contrast, as expected, the formulation F1 requires the use of five elements per blade to match the analytic solution. In Fig. 11b the deformed configurations are shown at t=100 s.

#### 4.2.3 Numerical results: flexible case

The goal of this case is to test the proposed formulation for a highly-flexible propeller. To do so, a Young modulus E=2.1 kPa is considered. This local flexible behavior plays a key role in applications such as the design of morphing wings [46], [43], [11], and represents a challenge for numerical methods.

**Figure 12:**
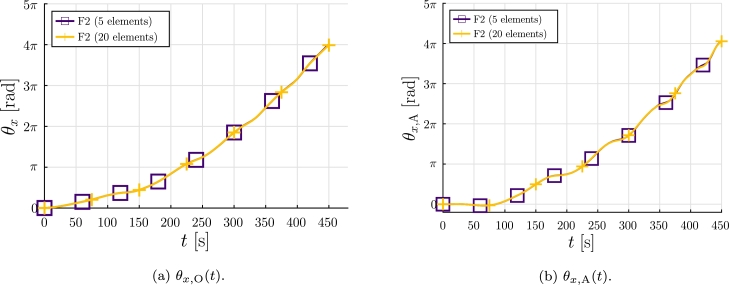
Example 2: Flexible case results of *θ*_*x*,A_(*t*) and *θ*_*x*,O_(*t*) rotations.

**Figure 13:**
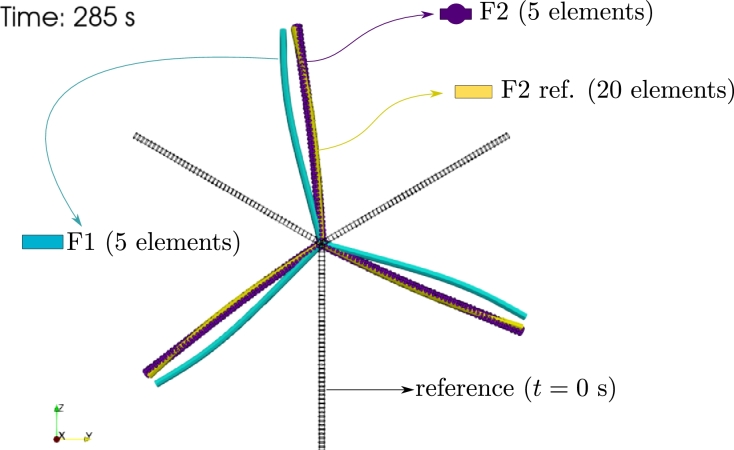
Example 2: Flexible case deformed configurations at time *t* = 285 s.

In this case, due to the bending deformation, the rotations of points O and A, shown in Fig. 10a, are considerably different. The numerical results obtained for the point O and point A using formulation F2 are presented in Figs. 12a and 12b, respectively. Formulation F1, using 5 and 20 elements, was not able to provide a numerical solution, hence the solutions were discarded. The deformed configurations obtained using F1 and F2 formulations at t=285 s are shown in Fig. 13.

The results let us conclude that, in this problem, for flexible elements with rotations larger than 2*π* and 20 elements per blade, formulation F1 is not able to provide a solution and formulation F2 converges for all time steps.

The results obtained let us conclude that the F2 formulation provides accurate results for large rotations and considerable bending deformations. We consider that the differences in performance between the formulations are due to the approach used for computing the inertial terms. In the following example, a non-zero aerodynamic pitch moment is considered, providing a more complete comparison.

### 4.3 Example 3: simplified cantilever blade

In this example a cantilever beam with an airfoil cross-section is considered. Realistic drag, lift and moment aerodynamic coefficients are used. The goal of this example is to compare the solutions obtained with F1 and F2 formulations, considering realistic aerodynamic coefficients.

#### 4.3.1 Problem definition

**Figure 14:**
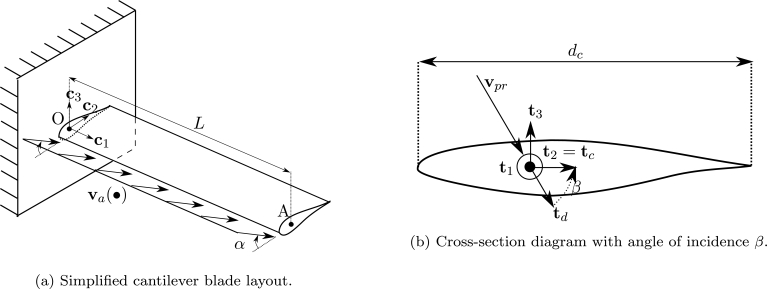
Example 3: Diagram and cross-section of the cantilever blade problem.

**Figure 15:**
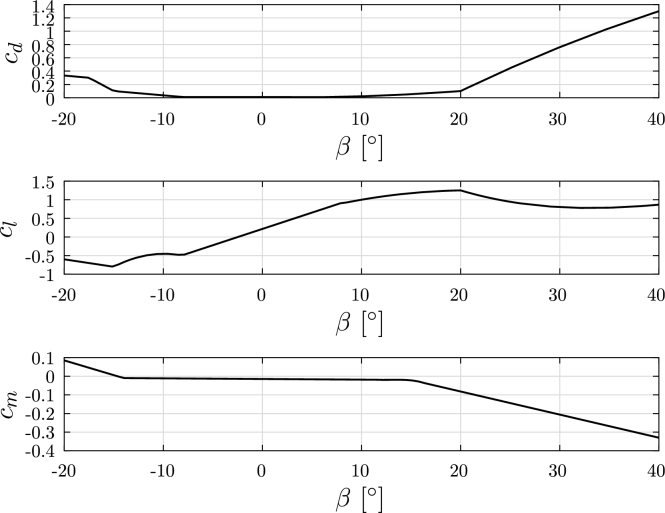
Example 3: Functions considered for drag (top), lift (middle) and moment (bottom) extracted from [36].

In this example a cantilever beam submitted to a fluid flow with varying direction, as illustrated in Fig. 14a. The cross-section of the beam is given by a NERL S809 wind turbine airfoil, with L=10 m and chord length of $d_{c}=1$ m [23]. The aerodynamic coefficient functions, obtained from [36], are shown in Fig. 15. The geometry of the problem is inspired on an specimen presented in [16] and for the material properties, equivalent Young modulus $E_{eq}=14$ GPa and shear modulus $G_{eq}=5.6$ GPa are adopted.

The flow velocity is uniform and its expression is given by Equation (42):

(42) $$v_{a}(x)=v_{m}\left(\cos(\alpha )c_{2}-\sin(\alpha )c_{3}\right)$$

with $v_{m}=30$ m/s and $\alpha \in [0^{\circ },40^{\circ }]$. The problem is solved considering a static analysis for each value of *α* considered. The change in *α* can be associated with a slow change in the pitch angle during the operation of a wind turbine.

#### 4.3.2 Numerical results

For the numerical resolution 10 co-rotational frame elements with F1 and F2 were used. The N-R method is used, considering $tol_{r}=5\times 10^{-7}$ and $tol_{u}=10^{-15}$. For the computation of the numerical solution, 4 Gauss integration points were considered for computing the aerodynamic forces.

**Figure 16:**
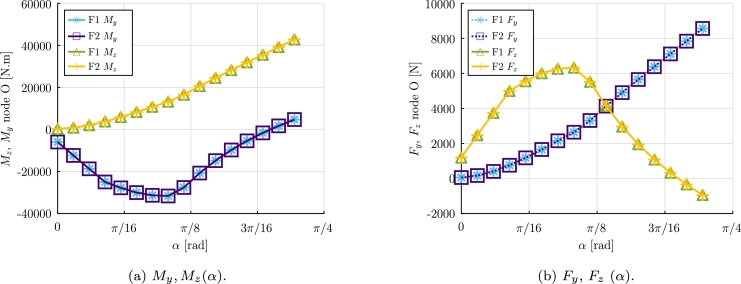
Example 3: Reaction bending moments *M*_*y*_(*α*), *M*_*z*_(*α*) and resultant shear forces *F*_*y*_(*α*) and *F*_*z*_(*α*) at node O.

**Figure 17:**
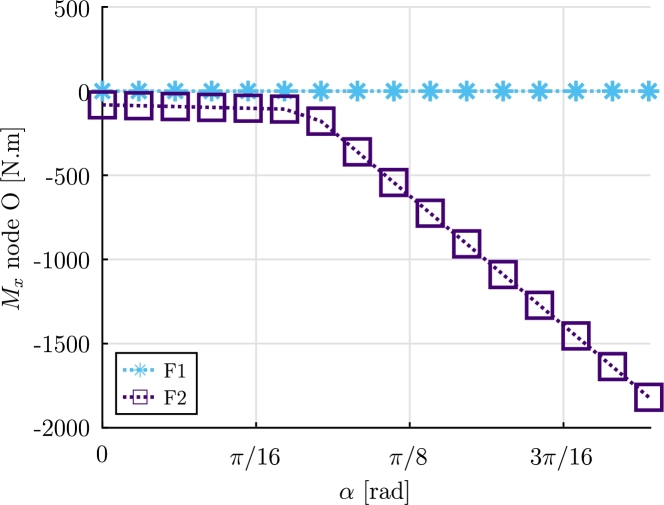
Example 3: Torsional moment *M*_*x*_(*α*) at node O.

The results obtained for the bending moments $M_{z}$, $M_{y}$, are presented in Fig. 16a. Additionally, the resultant shear forces $F_{z}$ and $F_{y}$ at node O, are shown in Fig. 16b. The torsional moments at point 0 ($M_{x}(\alpha )$), for both formulations, are illustrated in Fig. 17. The torsional moment at point 0 provided by F1 with $\alpha =40^{\circ }$ is 0.03 N m, while F2 provided a moment −1819 N m.

It can be observed the formulation F1 largely underestimates the torsional moment at point O. The results obtained let us conclude that F1 should not be used for the resolution of problems in which the torsional moment is relevant. On the other hand, formulation F2 provided appropriate results.

### 4.4 Example 4: simplified wind turbine

#### 4.4.1 Problem definition

In this example a flexible frame structure undergoing significantly large rotations is considered. For this, a simplified wind turbine model is developed, where the fundamental features of the real problem are present.

**Figure 18:**
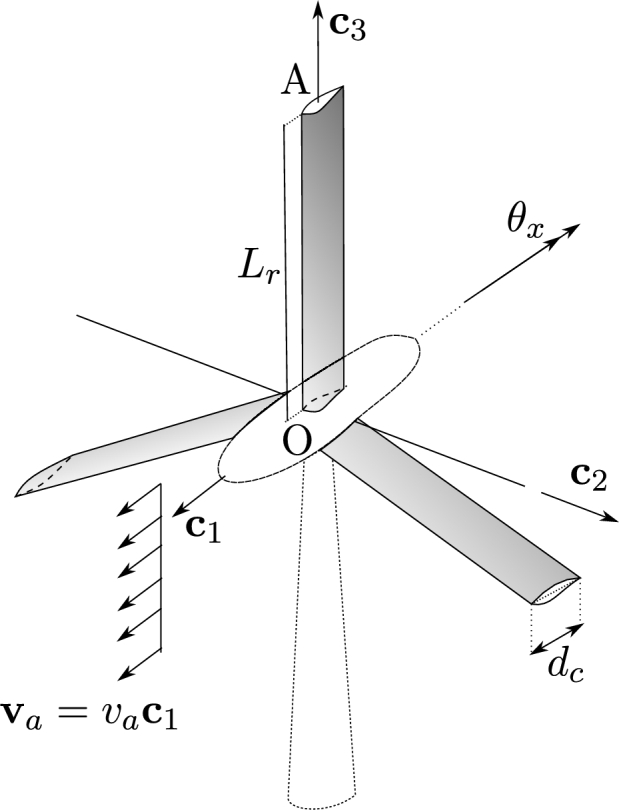
Example 4: Uniform wind turbine layout.

The problem consists in an idealized wind turbine as shown in Fig. 18. Each blade has a uniform NERL airfoil with the geometry and material properties presented in Section 4.3. The aerodynamic coefficients were extended to obtain values between -30^∘^ and 90^∘^ based on [22]. A uniform constant wind velocity $v_{a}=27\;c_{1}$ m/s is considered. The initial conditions are homogeneous both for displacements and velocities of the blades.

#### 4.4.2 Numerical results

The *α*-HHT numerical method is used with a time step $\Delta_{t}=0.01$ s and a final time $t_{f}=30$ s. The residual force and displacement tolerances are: $tol_{r}=10^{-5}$ and $tol_{u}=10^{-10}$. The spatial discretization of each blade is done using 30 aerodynamic co-rotational elements. The formulation F1 was not able to provide a numerical solution, while F2 provided the results shown.

**Figure 19:**
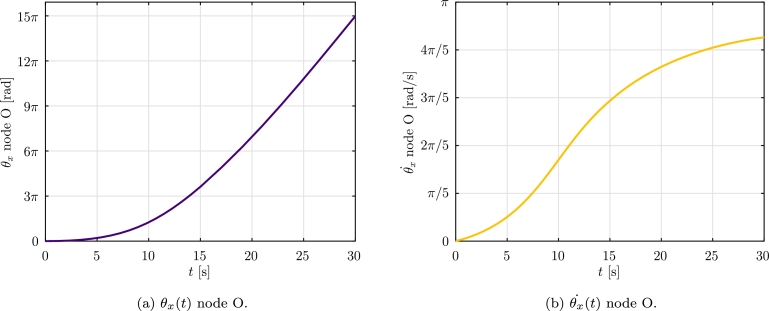
Example 4: *θ*_*x*_ angle and velocity rotation.

**Figure 20:**
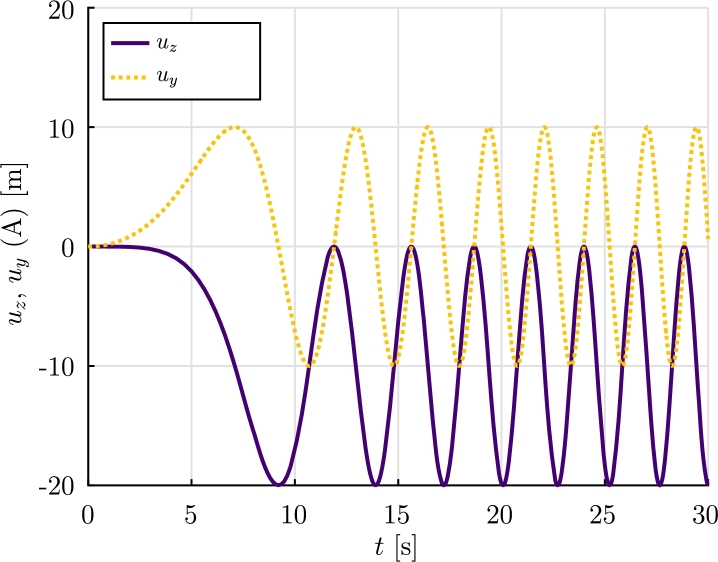
Example 4: Displacements *u*_*y*_(*t*) and *u*_*z*_(*t*) of node A.

The numerical results obtained for the rotation $\theta_{x}$ of point O, are shown in Fig. 19a, and the results of the angular velocity $\overset{˙}{\theta_{x}}(t)$ of point O, are shown in Fig. 19b. The horizontal $u_{y}$ and vertical $u_{z}$ displacements of node A are shown in Fig. 20.

The results let us conclude that formulation F2 is able to provide a numerical solution of the problem. Moreover it can be observed that, as expected, the angular velocity does not diverge and a quasi-stationary regime is reached.

## 5 Conclusions

In this article a new formulation for the numerical analysis of frame structures submitted to aerodynamic forces is presented. The methodology extends the application of the co-rotational approach, for computing the quasi-steady aerodynamic forces in the deformed configuration for large displacements and rotations. The co-rotational approach is used to compute aerodynamic, internal and inertial forces, providing a set of nonlinear governing equations. An aerodynamic stiffness matrix is added to the tangent matrix in the numerical procedure using a finite difference approach. Three formulations were considered: F1, which is considered equivalent to a previous work of the literature, and F2/F3 two variants of the proposed co-rotational formulation.

The results obtained using the formulations were presented and compared in four numerical examples. The numerical resolution procedures associated with the formulations were implemented in the open-source FEM library ONSAS. All the scripts used to generate the results are publicly available.

In Example 1, the reconfiguration of a cantilever beam problem submitted to drag forces was considered. The results obtained for the static case let us conclude that, for small deformations the three formulations match the reference solution. On the other hand for large deformations, formulation F3 is more robust than F1 and F2. For the dynamic analysis case the three formulations provide accurate results and formulation F3 requires more execution time. Finally, a numerical study on the Gauss integration of the aerodynamic forces is performed and, 4 Gauss integration points are selected as an adequate number.

In Example 2, a three-blade propeller submitted to a uniform wind flow undergoing large rotations was considered. Two cases were defined for the stiffness of the blades (rigid and flexible), and the analytic solution is presented for the rigid case. The results obtained let us conclude that, as expected, the proposed formulation F2 provides accurate results and requires a lower number of elements than F1. For the flexible case, the formulation F1 is unable to solve the problem, while formulation F2 provides adequate results.

In Example 3, a simplified wind turbine blade submitted to a fluid flow with uniform velocity and rotating direction was considered. Realistic drag, lift and pitch aerodynamic coefficients were considered based on reference literature and a static analysis was performed. The results let us conclude that, for this static analysis case, formulation F1 is not able to provide a proper value for the torsional moment, while formulation F2 provides adequate results. It can be inferred that formulation F2 would be a preferred option for potential use in Engineering design problems.

In Example 4, the dynamic analysis of a realistic wind turbine submitted to a fluid flow with uniform velocity was considered. The results obtained let us conclude that, formulation F2 is able to provide the expected behavior, while formulation F1 is not able to solve the problem for the parameters considered.

The proposed co-rotational formulation represents a simple yet accurate tool for simulating flexible structures submitted to fluid loads.

Several research directions can be considered for future work. The proposed formulation could be validated using results provided by other more computationally demanding FSI approaches. Moreover, including eccentric aerodynamic and mass centers could be the next step if real world problems are to be solved. Finally, extending the aerodynamic forces model to reproduce unsteady phenomena, such as flow-induced vibrations can also be considered.

## CRediT authorship contribution statement

**Mauricio C. Vanzulli:** Conceived and designed the experiments; Performed the experiments; Analyzed and interpreted the data; Contributed reagents, materials, analysis tools or data; Wrote the paper.

**Jorge M. Pérez Zerpa:** Conceived and designed the experiments; Analyzed and interpreted the data; Contributed reagents, materials, analysis tools or data; Wrote the paper.

## Declaration of Competing Interest

The authors declare that they have no known competing financial interests or personal relationships that could have appeared to influence the work reported in this paper.

## Data Availability

Data associated with this study has been deposited at https://github.com/mvanzulli/SourceCode_Manuscript_2204.10545.

## Footnotes

1 https://github.com/mvanzulli/SourceCode_Manuscript_2204.10545.

2 https://github.com/lm2-poly/Reconfiguration-Beam.
